# Present Situation of Cystic Echinococcosis in Africa: A Narrative Review

**DOI:** 10.3390/vetsci13060562

**Published:** 2026-06-06

**Authors:** Guo-Qiang Zhu, Abbas Abdelkerim Adam Daoud, Gui-Xiang Teng, Zheng-Yang Niu, Xin Feng, Xiao-Pei Luo, Constantine Uwaremwe, Nigus Abebe Shumuye, Hussam Askar, Hong-Bin Yan, Li Li

**Affiliations:** 1 School of Biological and Pharmaceutical Engineering, Lanzhou Jiaotong University, Lanzhou 730070, China; 2 State Key Laboratory of Animal Disease Control and Prevention, College of Veterinary Medicine, Lanzhou University, Lanzhou Veterinary Research Institute, Chinese Academy of Agricultural Sciences, Lanzhou 730046, China; 3 Department of Veterinary Clinical Medicine and Epidemiology, College of Veterinary Sciences, Mekelle University, Kalamino Campus, Mekelle P.O. Box 2084, Tigray, Ethiopia; 4 Zoology Department, Faculty of Science, Al-Azhar University, Assiut 71524, Egypt

**Keywords:** cystic echinococcosis, prevalence, risk factors, Africa

## Abstract

Cystic echinococcosis (CE) caused by the parasite *Echinococcus granulosus* is a zoonotic disease of global concern. Because most African countries have not yet conducted systematic studies on CE, it is difficult to determine its exact status. However, as with any parasitic zoonotic disease, the first step in developing a comprehensive management and control strategy is to identify the host range, transmission risk factors, and national and regional epidemiological data. This review evaluated and summarized articles on CE retrieved from the PubMed and Africa Journal Online (AJOL) databases. Inclusion criteria were studies (including case reports) focused on the prevalence of CE in animals and humans between 1970 and 2025. This review revealed that although numerous early studies on CE exist, only a few were conducted in Africa from 1970 to 2025. Furthermore, data regarding population genetic composition and the role of wildlife in CE transmission remain scarce. This review systematically evaluated the prevalence and transmission of CE in Africa and identified priority areas for surveillance and control.

## Simple Summary

Cystic echinococcosis (CE) is a zoonotic disease caused by the larval stage of *Echinococcus* *granulosus*. It poses a significant public health and economic burden in many African countries, particularly among pastoral communities where close contact with dogs and livestock is common. This review summarizes the current state of knowledge on CE in Africa, including its prevalence in humans and animals, geographical distribution, risk factors, diagnostic methods, treatment options, and control strategies. By highlighting knowledge gaps, such as the lack of molecular data and limited surveillance in Central Africa, this review aims to guide future research and support the development of effective prevention and control measures across the continent.

## 1. Introduction

Cystic echinococcosis (CE) is a zoonotic disease caused by the larval stage of *Echinococcus granulosus* (*E. granulosus*). The adult worms parasitise in the small intestine of the definitive hosts (particularly domestic dogs and other wild canids such as wolves and foxes) and excrete eggs via their faeces. Intermediate hosts (mainly including sheep, cattle, donkeys, and camels, but also humans) are infected by ingesting these eggs. In the intermediate hosts, the eggs evolve into larvae (hydatid cyst), which develop cysts at the lesion site and consequently cause CE. The cysts are generally classified as viable or non-viable. Viable cysts typically contain clear fluid and a small amount of calcified material, while non-viable cysts are primarily composed of calcified material. Viable cysts are the main type that causes CE [1].

Historically, *E. granulosus* was the sole species known to cause CE. To reflect diversity, 10 strains (G1–G10) have been identified, each linked to an important intermediate host for transmission, such as the sheep strain G1 or the camel strain G6. Ongoing research continues to reassess this classification, but currently, CE can be caused by *E. granulosus* (G1–G3), *Echinococcus felidis* (*E. felidis*) (the “lion” strain), *Echinococcus equinus* (*E. equinus*) (G4), *Echinococcus ortleppi* (*E. ortleppi*) (G5), and *Echinococcus canadensis* (*E. canadensis*) (G6–G10) [2]. Most studies have not distinguished these differences, resulting in limited information about the various species, strains, hosts, and clinical impacts. Many human cases are believed to be caused by *E. granulosus*. Except for *E. felidis* and some wild canid/felid-associated strains, most *E. granulosus* strains involve domestic dogs as definitive hosts [3].

*Echinococcosis* is present worldwide and endemic in certain regions, including the Mediterranean coast, Central Asia (including the Tibetan Plateau), North and East Africa, Australia, and South America [4]. Nomadic populations keep dogs for various reasons: herding, guarding, feeding, warmth, and grooming. Close contact between humans and dogs, water scarcity, and inadequate sanitation collectively foster favorable conditions for the transmission of echinococcosis. This neglected tropical disease suffers from a critical lack of funding for research and clinical treatment, given its impact [5].

This article describes the risk factors, diagnosis, treatment, and prevention of CE, particularly in North, East, West, and Southern Africa [4]. The epidemiology of echinococcosis in Africa remains poorly understood [6]. Our review aimed to summarise available data on CE in Africa and provide the most comprehensive description of current knowledge of the disease.

Overall, this review will provide a theoretical framework for understanding CE research and epidemiology in Africa, providing important implications for disease prevention and control strategies in Africa.

## 2. Methods

The AJOL, Google Scholar, PubMed, ScienceDirect, Scopus, and Web of Science databases were used to conduct a narrative literature review. Two different Medical Subject Headings (MeSH) terms were used in this narrative review: (i) “human” AND “Africa” AND “*E. granulosus*” OR “echinococcosis”; (ii) “Africa” AND “*E. granulosus*” OR “dog tapeworm”. The search period from 1 January 1970 to 31 December 2025 was chosen to compare studies from different decades. By searching for information sources on CE, more studies that lacked sufficient online information were found in CE research in Africa. Studies were included if they reported original data on human or animal CE prevalence in Africa, published 1970–2025 in English or French. Reviews, letters, editorials, and case reports without prevalence data were excluded. Risk-of-bias (ROB) was assessed as low risk (LR) or moderate risk (MR).

## 3. Cystic Echinococcosis Research in Africa

### 3.1. Echinococcus granulosus in West Africa

**Figure 1:**
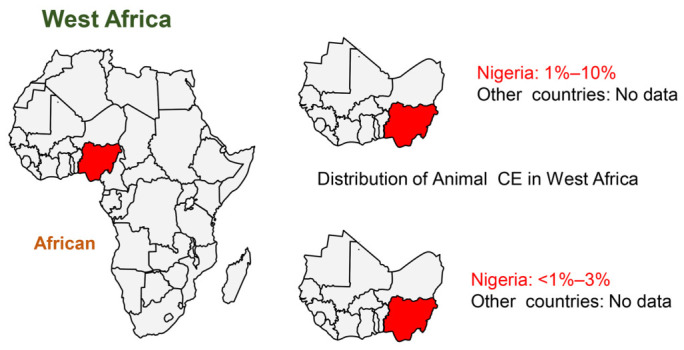
Geographical distribution of published studies on cystic echinococcosis in West Africa.

CE is generally uncommon in West Africa, and epidemiological studies have been conducted only in Nigeria (involving both people and livestock) and in Burkina Faso (limited to livestock) (Figure 1). Nevertheless, sporadic human cases of CE have also been reported in Senegal, Niger, and Ghana [7].

A limited case series from Niger (n = 32) has been reported, which demonstrated female predominance (n = 20), consistent with epidemiological patterns observed elsewhere in Africa [6]. Notably, this cohort presented a high proportion of hydatid cysts with extrahepatic localization, and the prevalence of extrahepatic echinococcosis was further quantified. In a parallel livestock survey, infection with *E. granulosus* was identified in 22% of 513 camels examined [8]. A single case of human cystic echinococcosis (HCE) has been reported from the Central African Republic, with molecular characterization identifying the causative agent as the G6 genotype (camel strain). Notably, the study noted an absence of camels in the Central African Republic. Moreover, no cases of CE have been documented in sheep, and another recognized host for the G6 strain [9]. Consequently, the transmission dynamics and maintenance of the *Echinococcus* life cycle within the Central African Republic remain to be fully elucidated.

A retrospective survey of bovine CE in Burkina Faso has been documented. The study identified hydatid cysts in 10 animals across multiple species from an estimated population of approximately 1,000,000 [10]. While no epidemiological data on human infection are available from the country, CE is not considered a major public health concern in Burkina Faso. Furthermore, genetic characterisation of the *Echinococcus* strains circulating in this region remains lacking. Nigeria is among the more extensively studied countries in West Africa regarding CE. While human echinococcosis is considered uncommon, it is likely underdiagnosed [11]. A serosurvey using a complement fixation test among hospital staff and patients in the country’s northern and southwestern regions detected antibodies to an echinococcal antigen in 0–53% of 176 individuals. However, corresponding clinical data to confirm active infection in these seropositive individuals were not available [12]. A review of hospital records spanning three regions of Nigeria identified a single confirmed case of CE out of over 500,000 records [13]. However, the researchers questioned the accuracy of this result because four additional cases were identified in a single region of northern Nigeria during the study period. The highest prevalence of *E. granulosus* infection in livestock was reported in cattle from the Niger Delta and northern Nigeria [13]. In the Niger Delta, pigs constituted the most frequently infected species, with 55.9% of 320 infected pigs exhibiting fertile cysts. In contrast, northern Nigeria reported a high prevalence of infection primarily in camels, with 55.5% of 3598 infected camels harboring fertile cysts [6]. Conversely, CE appears uncommon in the central and eastern regions, except in the north-central zone, where 11% of 1800 sheep were infected.

Intriguingly, the majority of reported HCE cases in Nigeria are concentrated in the northern regions [14]. The observed disparities in infection rates and cyst fertility across different livestock species and geographical zones suggest the potential circulation of distinct *E. granulosus* genotypes within Nigeria. These genotypes may exhibit varying zoonotic potential, which could explain the paradoxically low human infection prevalence despite the documented high burden of the disease in domestic animal reservoirs.

### 3.2. Echinococcus granulosus in East Africa

**Figure 2:**
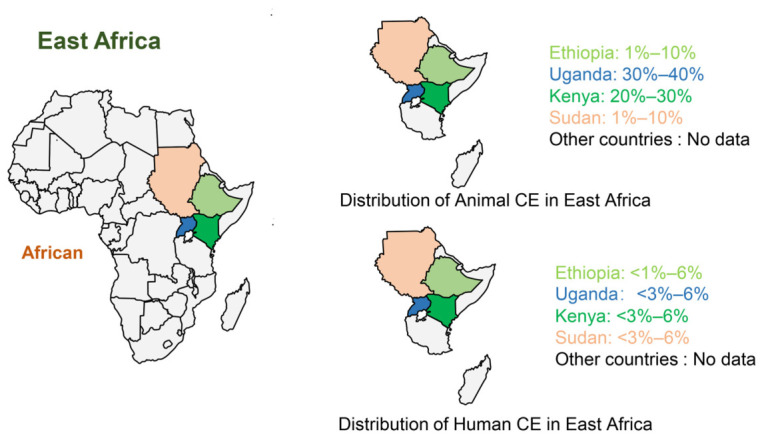
Geographical distribution of published studies on cystic echinococcosis in East Africa.

Most research on CE has been conducted in East Africa, specifically in Kenya’s Turkana and Maasai areas, as well as bordering districts in Ethiopia, Sudan, and Uganda (Figure 2) [15].

In Sudan, the distribution of CE is highly heterogeneous. An ultrasound study conducted in the far southeast, near the border with Turkana, identified a high-prevalence focus, with rates reaching 2% in humans. In contrast, human cases elsewhere in the country appear sporadic; however, a case series in Khartoum noted that pulmonary symptoms are common, accounting for 17 of the 38 cases. In animals, the infection was first documented in dogs in 1962, with a prevalence of 86.4% for *E. granulosus* [16].

Data on CE in cattle are available from central, western, and southern regions of Sudan. In central Sudan, prevalence rates range from 20% in cattle to 55.6% in camels [17]. Western Sudan shows the highest prevalence among camels (61.4% of 565 animals, with 74% cysts) and sheep (11.9% of 9272 animals, with 19% cysts). Cattle in this region have a lower infection rate (5.2% of 4318 animals), but cyst prevalence is notably high (75%) [17]. Additionally, 1.9% of 5523 goats are infected with *Echinococcus,* and 33% of the cysts are fertile. Southern Sudan reports much lower prevalence rates in cattle (7.1% of 325), sheep (2.7% of 295), and goats (7.1% of 42) than in the west [17].

In Ethiopia, before ultrasound and modern serological tests were introduced as routine diagnostic tools, Fuller and Fuller showed that the Dassanetch and Nyangatom peoples in the southwest of the country had a prevalence of CE of up to 5% based on clinical examination findings, and over 5% if hydrodynamic skin tests were considered [18]. The Dassanetch and Nyangatom, who inhabit the same geographical region as the Turkana people in north-western Kenya, share similar customs, including the use of dogs for cleaning. By contrast, an ultrasound survey conducted among the Hamar people in southwest Ethiopia revealed a significantly lower prevalence rate (0.7% out of 990 individuals) than among the Dassanetch and Nyangatom [19]. In a case series from central Ethiopia involving 72–234 patients examined over 10–15 years at Addis Ababa hospitals, researchers found no female predominance, in contrast to patterns observed elsewhere. The incidence of CE was nearly equal in the lung and liver, with each accounting for approximately 40% of cases [19].

CE is widespread in Kenya, particularly among Turkana and Maasai communities in the northwest and south. Both are nomadic pastoralists who herd large numbers of livestock, such as sheep, goats, cattle, donkeys, and camels (Turkana). A serological survey found a 16.4% prevalence among recently settled Turkana community [20]. Another survey identified regional differences within Turkana, with 9.4% prevalence in the north and 2.1% in the south. A control group from other Kenyan regions showed virtually no cases. Overall, the prevalence in Turkana was 5.6% [21].

In Uganda, only a single study has documented CE in both humans and cattle. A six-year retrospective review of the National Pathology Service identified 23 human cases, with a noted female predominance. While the majority of cases (n = 12) were imported from Sudan, all 10 indigenous cases originated from the northern and northeastern provinces, adjacent to southern Sudan and the Turkana area of northern Kenya. It should be noted that in the Karamoja district, the area closest to Turkana and the source of five of the ten Ugandan cases, 20% of cattle were infected with *Echinococcus* [22]. In the other two districts (Acholi and Lango), where human cases were reported, the disease prevalence in cattle was 1%. In another county (Teso) to the south of the counties where human cases were reported, a 10.5% prevalence was observed in cattle fields [23]. Approximately two-thirds of dogs in Moroto County were infected with an undescribed strain of *E. granulosus*. The results of a survey carried out in Queen Elizabeth National Park, western Uganda, showed that a high proportion of resident lions were infected with *E. felidis*, *E. felidis* and *E. granulosus* cysts were recognized in warthogs from the same site [24].

### 3.3. Echinococcus granulosus in Southern Africa

**Figure 3:**
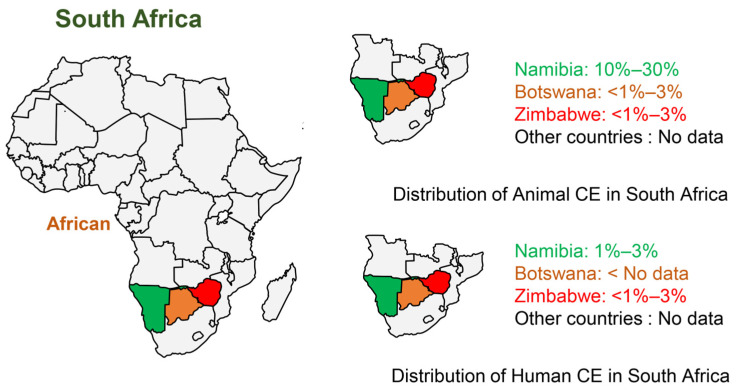
Geographical distribution of published studies on cystic echinococcosis in South Africa.

Research on echinococcosis in southern Africa, particularly in South Africa and Zimbabwe, has advanced substantially globally (Figure 3). Most epidemiological data on infection prevalence in intermediate livestock hosts are derived from abattoir surveys, which often lack traceability of the animals’ geographical origin. Furthermore, while certain demographic and environmental risk factors for human infection are recognized nationally, systematic surveillance data on human echinococcosis remain largely unavailable.

This section focuses on the epidemiology of HCE in South Africa, with 162 cases recorded in the country [25]. Most researchers have focused on the unusual symptoms and complications of CE, such as cysts in the central nervous system, spine, heart, and orbits, so these reports do not reflect the disease’s prevalence in South Africa. While comprehensive epidemiological studies are lacking, a consensus exists among reporting researchers that CE is encountered with notable frequency in certain regions of South Africa. Kayser reports around 20 cases annually in a hospital in the Eastern Cape province [26]. However, these clinical case reports typically offer limited insights into the specific risk factors for human infection. Consequently, the principal animal hosts, both intermediate and definitive, involved in the local transmission cycle of *E. granulosus* in South Africa remain poorly defined. In contrast, the disease appears rare in neighboring Zimbabwe, with only two documented cases reported to date [27].

In Zimbabwe, a slaughterhouse survey of bovine lungs revealed an infection prevalence of *E. granulosus* in 0.6% of 2000 examined specimens [28]. Notably, in contrast to typical findings in Europe, the vast majority (96.8%) of these cysts were fertile [29]. Some data on CE in animals are available from South Africa. In 1965, Verster and colleagues studied the prevalence of CE in cattle in the country’s abattoirs [30]. Subsequent reports indicate considerable geographical and species-specific variation. For instance, bovine infection rates have been documented to range from 1.2% to 13.8%, with the highest prevalence observed in the Eastern Cape and the lowest in the Karoo region [31]. However, these researchers also noted that prevalence rates increased with age in cattle, so that differences could be attributed to differences in the age of slaughtered animals rather than true differences in prevalence. For sheep, prevalence ranged from 0.8% in the Karoo to 2.3% in Mpumalanga. For goats, the prevalence ranged from 0% in the Western Province to 3.2% in the Eastern Cape, but the number of animals slaughtered was low [32]. In cattle, infection primarily affected the lungs, whereas in sheep and goats, it predominantly affected the liver. The liver was the most frequently affected organ in sheep and goats [33].

### 3.4. Echinococcus granulosus in Central Africa

CE is endemic in certain parts of Africa, where the disease is highly prevalent. Multiple genotypes that cause CE have been identified.

*E. granulosus* is highly common in dogs in parts of North and East Africa. Past studies have found a 25.8% prevalence of *Echinococcus* eggs in stray dogs in Libya and between 33 and 39.4% in stray dogs in the Turkana region of Kenya [34]. However, there is very limited data from Central Africa. A fecal microscopy survey conducted in northeastern Gabon (Ogouée-Ivindo) detected tapeworm eggs in 8.6% of canine faecal samples. However, morphological analysis failed to distinguish *Echinococcus* from other tapeworm species. Another study carried out in Libreville, Gabon, found tapeworm eggs in only 1% of canine faecal samples [35].

To our knowledge, no systematic studies on human echinococcosis are available in Central Africa. The most geographically relevant data come from Nigeria in West Africa, where a retrospective analysis of hospital records and a serological survey—both conducted in the 1980s—reported prevalence rates of 0% and 0.53%, respectively. [36]. A case report of a patient from the Central African Republic has been published, in which molecular characterisation revealed the presence of the Canadian strain of *Echinococcus* G6 [9]. Additional evidence from Central Africa is limited to isolated case reports. These include a case of splenic *E. granulosus* cysts in a patient from Yaounde, Cameroon; a case in a refugee from Niger diagnosed in Italy; and an earlier report describing a European man residing in the Democratic Republic of Congo [37]. Apart from these case reports, there are no systematic data from Central Africa.

### 3.5. Echinococcus granulosus in North Africa

**Figure 4:**
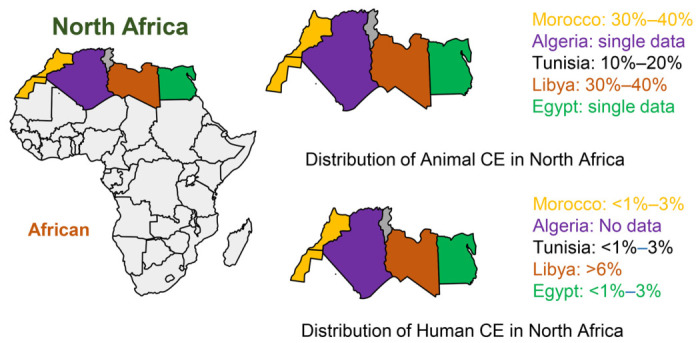
Geographical distribution of published studies on cystic echinococcosis in North Africa.

Previous and recent reports have described the prevalence of *E. granulosus* in dogs and livestock, as well as human infection, across all Arabic North African countries, including Algeria, Egypt, Libya, Morocco, and Tunisia [38]. Additionally, the map of North Africa (Figure 4) shows the number of reports of Echinococcus infections; most originate from Tunisia, Morocco, and Libya. In all countries where camels have been reported as intermediate hosts, camels are considered locally important for maintaining the life cycle.

CE is a public health problem in Egypt. Veterinarians recognize the presence of CE in slaughtered livestock, but there is little information on the prevalence of CE infection in humans [39]. The prevalence of CE in Egyptian sheep and camels is 1.03% and 31.00%, respectively. A survey of animals slaughtered for cysticercosis in two Egyptian abattoirs (Ismailia and Cairo governorates) revealed the following prevalence rates: 18.09% in camels, 4.08% in sheep, 1.02% in goats, 0.095% in cattle, and 0.046% in buffaloes. The seropositivity rate for *Echinococcus* antibodies was 26/426 (6.1%) in human sera. Therefore, *Echinococcosis* does not represent a major public health problem in Egypt. However, sheep play an important role in its transmission because of the high fertility of cysts compared to other intermediate animal hosts; the risk cycle is sheep-human-dog [40].

CE, caused by *E. granulosus,* remains a notable zoonosis in Libya. Necropsy results showed that *E. granulosus* was present in 25.80% and 21.60% of stray dogs, respectively [41]. The infection prevalence varied from 1.70% to 33.40% in sheep, 1.00% to 13.90% in cattle, 1.40% to 40.0% in camels, and 0.0% to 18.0% in goats. The incidence of surgically confirmed CE in eastern Libya was estimated at a minimum of 4.02 cases per 100,000 people, with females significantly more affected than males [42]. Both ultrasound and serological tests indicate that about 1.60% of northern Libya’s population is infected.

*Echinococcosis* is highly endemic in Morocco, with studies indicating a widespread prevalence in agricultural and pastoral regions among both humans and domestic animals. The infection rate in dogs is 33.00%, while it is 9.90% in sheep, 3.20% in goats, and 42.00% in cattle [38]. Human infection rates vary from 3.60 to 15.80 cases per 100,000 individuals. An ultrasound survey of 11,612 people in Morocco found a 1.10% infection rate for CE. Additionally, 77.40% of those screened via ultrasound also tested positive for *E. granulosus* [43,44].

A survey of helminth infections in stray dogs was conducted in the Testour and Bouzid regions of Tunisia. The results showed that 21.0% of the dogs killed were infected with *E. granulosis* [45]. The study also confirmed the role of small ruminants, especially sheep, as intermediate hosts in the area. In Kebili, no encapsulated vesicles were found in 103.0 camels, whereas 19.0 camels (10.10%) out of 188 in Ben Guelden were infected with *E. granulosus* [46]. The annual incidence of CE in Tunisia is estimated at around 15.0 cases per 100,000 people [47]. However, Tunisia has not yet conducted a national survey on the prevalence or annual incidence of CE, nor on its health impact. The rates of surgical cases from reported instances remain unknown [48].

## 4. Prevalence of Human Cystic Echinococcosis in Africa

### 4.1. Human Cystic Echinococcosis

HCE is common in many African countries, but data on its incidence, prevalence, and overall impact are limited. Nonetheless, some surveillance reports from different African nations exist, along with a standardized reporting system.

This review found a prevalence of 1.7% in Africa, lower than in Asia (2.2–6.0%) [49,50] and Latin America (4.7–7.1%) [51]. Conversely, the rate was higher than in European countries like Italy (0.2%) [52] and Slovakia (0.6%) [53]. Such differences may be associated with regional variations in free-range dog populations and dog ownership, which contribute to environmental contamination. Potential contributing factors also include household slaughter of livestock, feeding raw offal to dogs, and environmental and climatic conditions (e.g., temperature, rainfall, humidity) that influence parasite egg survival.

Our analyses identified regional variations in HCE prevalence, with East Africa showing the highest rates, followed by North Africa. Table 1 presents the characteristics of the HCE studies. The majority of these studies (53.9%) originate from Northern Africa [20]. These findings align with existing reports highlighting the disease’s widespread occurrence among nomadic groups in East Africa [6,54]. Additionally, similar regional differences in HCE prevalence were observed across other continents, such as Asia and Latin America [51].

**Table 1:** A list and description of published research on human cystic echinococcosis conducted in Africa.

Country	Study Year	Sample Number	Cases	Prev (%)	ROB	Reference
C/Africa						
Gabon	2014	348	0	00	MR	[55]
E/Africa						
Ethiopia	2008	24,840	26	0.11	LR	[56]
Kenya	1991	537	87	16.35	MR	[57]
Mozambique	2011	600	103	17.29	MR	[58]
South Sudan	2012	609	3	0.65	MR	[59]
Tanzania	2012	344	38	11.29	MR	[60]
N/Africa						
Egypt	2006	20	3	14.28	MR	[61]
Libya	2008	299	26	8.00	LR	[62]
Morocco	2014	5366	101	1.91	LR	[63]
Tunisia	2004	2154	25	2.07	MR	[64]
Sudan	2017	304	19	6.55	LR	[17]
S/Africa						
South Africa	1995	235	25	11.01	MR	[65]
W/Africa						
Nigeria	1986	175	1	1.20	MR	[66]

E/Africa eastern Africa, MR moderate risk, N/Africa northern Africa, LR low risk, S/Africa southern Africa, ROB risk of bias, W/Africa western Africa.

These studies used three methods to identify HCE infection: serologic tests, ultrasound, and “other methods” (such as autopsy, surgical procedures, or combined techniques). Serological assays showed the highest apparent prevalence, which may be partially attributed to their high sensitivity (allowing detection of small cysts missed by ultrasound). However, this finding must be interpreted cautiously, as seropositivity does not distinguish active infection from past exposure, and cross-reactivity can lead to overestimation of true prevalence [67]. Moreover, antibodies to hydatid cysts can remain long after the cysts are removed, even following chemotherapy or surgery [68]. Ultrasound is the preferred diagnostic tool for HCE, as it helps determine the location, number, and size of cysts.

Our study revealed a 1.0% increase in CE cases over 51 years (1970–2021), with an initial 0.3% decline from 1988 to 2004, then a 1.3% increase from 2005 to 2021. These patterns indicate that disease control efforts in Africa might be irregular.

### 4.2. Animal Cystic Echinococcosis in Africa

In Africa, dogs are now recognized as true hosts for *E. granulosus*. However, only a few studies meeting the inclusion criteria have been conducted to determine its prevalence and distribution. The metacestode of *E. granulosus* is associated with zoonoses, HCE, and notable economic losses in the meat industry.

The 16.9% prevalence of canine *E. granulosus* infection in Africa aligns with global data. For instance, studies in Asia report rates between 7.3% and 48% [69], in Latin America from 9.3% to 42.3% [70,71], and in Australia from 3.16% to 50.7% [71], all confirming the worldwide presence of canine *E. granulosus* infection. These infections can contaminate the environment, increasing the risk of transmission to intermediate hosts such as domestic ruminants and sustaining the disease cycle. In Africa, regional variations exist in the prevalence of canine *E. granulosus* infection—highest in North Africa, followed by East Africa, which shows a similar pattern. Table 2 presents the characteristics of the reported EGI in dogs. These differences likely result from factors such as wild dog intrusion into urban areas [72], the density of intermediate hosts, and climate and environmental influences on egg development and survival [73].

**Table 2:** A list and description of published research on *Echinococcus granulosus* infections in African dogs.

Country	Study Year	Sample Number	Cases	ROB	Prev (%)	Reference
C/Africa						
Gabon	2014	128	0	MR	00	[55]
E/Africa						
Ethiopia	2010	43	14	MR	34.08	[74]
Kenya	2013	1620	177	MR	10.97	[75]
Uganda	2013	260	31	LR	12.25	[76]
Zambia	2006	539	0	MR	0.00	[77]
N/Africa						
Algeria	2006	119	21	MR	18.32	[78]
Egypt	2006	49	7	MR	15.00	[61]
Libya	2001	49	28	MR	57.00	[79]
Morocco	2010	253	103	MR	40.93	[80]
Sudan	2004	83	40	MR	48.80	[81]
Tunisia	2014	287	31	MR	11.10	[82]
S/Africa						
South Africa	1978	1062	9	MR	0.93	[83]
W/Africa						
Mali	2010	117	1	MR	0.84	[84]
Nigeria	2019	26,843	2485	MR	9.25	[85]

E/Africa eastern Africa, MR moderate risk, N/Africa northern Africa, LR low risk, S/Africa southern Africa, ROB risk of bias, W/Africa western Africa.

Other factors may include differences in the number of stray dogs across regions. Serological methods, especially Enzyme-Linked Immunosorbent Assay (ELISA), revealed the highest prevalence of HCE. Botulinum infection is likely attributed to this method’s high sensitivity and specificity, as well as its capacity to differentiate between active and convalescent infections [57]. Interestingly, serological techniques also showed the highest prevalence of HCE infection. Among the three methods used for *Echinococcosis* detection, ELISA, microscopy, and polymerase chain reaction (PCR), PCR had the lowest detection rate at 14.5%. Besides reporting prevalence, these PCR methods identified genotypes such as *E. granulosus* (s.s.) (G1–G3) in Sudan, *E. canadensis* (G6–G9) in Sudan and Mali, as well as *E. ortleppi* (G5) and *E. felidis* (lion strain) in Kenya [75]. A subgroup analysis by study year showed an initial 6.7% increase in incidence from 1991 to 2006, followed by a 7.4% decrease from 2007 to 2021, suggesting challenges in current pathogen control efforts in Africa. The prevalence of *E. granulosus* decreased with larger sample sizes, aligning with the findings from the HCE study.

## 5. Risk Factors

In endemic regions worldwide, *E. granulosus* infection has been linked to resource-limited settings, pastoral lifestyles, specific grazing practices, and socioeconomic and behavioral factors [86,87]. Additional contributors include sources of drinking water, environmental changes that favor egg survival, cross-border animal movements, livestock trade, the high presence of stray dogs, frequent dog–human contact, poor slaughterhouse conditions, and improper waste disposal [56,88]. In Nigeria, besides these factors, the age of cattle plays a significant role, with animals [89] like camels being more susceptible—often slaughtered after 8–10 years, increasing infection risk [90]. Recently, temperature fluctuations, with some states reaching 44 °C, have been identified as potential factors affecting disease prevalence by drying out eggs [91]. Moreover, given the scarcity of studies that pinpoint local drivers of human and animal disease transmission in Africa, conducting such research is essential to understand regional transmission dynamics.

## 6. Diagnostic Tests

Diagnostic methods for detecting human hepatic echinococcosis include ultrasound, computed tomography (CT), and magnetic resonance imaging (MRI) [92]. Ultrasound (US) is the preferred method for detecting hepatic and extrahepatic echinococcal cysts; the sensitivity of this technique ranges from 92% to 97% [93]. CT confirms the diagnosis by detecting the presence of cysts and calcifications in the cyst wall; the sensitivity of CT is between 90% and 96% [93]. MRI reveals the characteristics of the hydatid cyst; US imaging and CT also contribute to the preoperative diagnosis. CT (as well as MRI) is essential for diagnosing cerebral hydatid cysts [94]. Chest CT with contrast is the preferred diagnostic modality for the diagnosis of pulmonary hydatid cyst, and the “air bubble” sign (a relatively new radiologic marker) is highly sensitive (85.6%) and specific (96.5%) in determining the diagnosis of a ruptured and infected hydatid cyst [95,96].

In animals, ultrasound-guided fine-needle aspiration effectively distinguishes abscesses from tumors, cysts, and other lesions. When pulmonary lesions are present, pathogens can often be identified in sputum or bronchial washings [97]. While histopathological examination may be enough, techniques such as immunohistochemistry or PCR can further assist in diagnosis. Specifically, *Echinococcus* species can be distinguished by analysis of cyst tissue using PCR, followed by sequence analysis or restriction fragment length polymorphism (RFLP) analysis [98].

## 7. Treatment

Treatment options for CE include chemotherapy, percutaneous radiation, and surgery. Historically, surgery was the sole treatment option, as it could completely eradicate the parasite. Over time, various surgical techniques have been developed, from cystectomy through increasingly invasive procedures to liver transplantation as the final option [99]. More invasive surgeries are associated with higher complication risks, including infection and bile leakage. However, they present a lower recurrence rate of active disease compared to conservative treatments.

Medical treatment using albendazole and mebendazole proves effective for patients with hydatid cysts in the liver and lungs. Combined use of albendazole and praziquantel has proven effective in treating such cases, demonstrating superior efficacy to monotherapy with either agent. Additionally, flubendazole, whether used alone or with ivermectin, is also effective against hydatid cysts [100].

Over the past two decades, ultrasound-guided percutaneous procedures have become the preferred treatment, gradually replacing surgery. Techniques such as percutaneous puncture, aspiration, injection, and respiration (PAIR), along with their modification, and percutaneous evacuation of cyst contents (PEVAC), are safe and effective alternatives to surgical intervention [101]. Drug therapy alone can be used to suppress early-stage disease or alongside surgical and minimally invasive procedures like puncture, injection, and aspiration for active large cysts. Despite the availability of drugs, interventional techniques remain the primary treatment for CE in Africa [102]. This is mainly because of limited resources to supply expensive medications and laboratory facilities to monitor side effects. Additionally, the nomadic lifestyle of many patients further complicates treatment.

## 8. Conclusions

This narrative review systematically synthesizes the current data on the prevalence, risk factors, and current prevention and control strategies for CE in Africa. Variations in prevalence rates across regions highlight the influence of ecological, socioeconomic, and health-related factors affecting disease [103]. The findings reveal substantial regional disparities in CE prevalence, with East Africa exhibiting the highest burden, followed by North Africa. Serological surveys consistently report higher apparent prevalence than ultrasound or clinically confirmed cases, largely due to differences in diagnostic performance and the detection of past exposure alongside active infection. Socioenvironmental factors, including free-range dog density, livestock management practices, and climatic conditions, are likely associated with geographic variations in disease distribution, although definitive causal evidence remains lacking. CE and alveolar echinococcosis (AE), caused by *Echinococcus multilocularis,* are significant forms of human echinococcosis. Although once thought to be rare in Africa, recent evidence indicates that AE might be more widespread than previously believed [104]. New studies have documented indigenous human infections in North Africa, including Tunisia, and identified potential transmission hotspots in East Africa, encouraging further research. AE tumor-like growth pattern and high mortality without treatment make its surveillance crucial, comparable to CE in endemic regions [105]. Comparative research between AE and CE offers valuable insights: CE models pastoral transmission dynamics, while AE enhances understanding of wildlife-related transmission cycles. Both are vital for comprehending the epidemiology, pathogenesis, and control of echinococcosis in Africa. Several critical limitations characterize the existing evidence base: marked heterogeneity in study design and diagnostic methods, limited molecular data on *Echinococcus* species/genotype diversity, sparse surveillance in Central Africa, and insufficient understanding of wildlife involvement in transmission cycles [106]. These gaps hinder precise assessment of the true burden and regional transmission dynamics. A comprehensive approach that considers multiple factors is essential for developing targeted prevention and control strategies.

The prevalence of CE in Africa is complex, with varying prevalence rates and risk factors across different regions [19]. A comprehensive prevention and control strategy is crucial to containing the disease’s spread. This strategy includes the rational use of anthelmintics to protect animal health. Standardize meat inspections in slaughterhouses to prevent diseased meat from entering the market. Strengthening the management of stray dogs to reduce environmental pollution [107]. Improving environmental hygiene to block transmission routes for pathogens and conducting large-scale screenings of people in hyper-endemic areas to enable early detection and treatment. In the future, research on CE in Africa should be enhanced. Future research priorities should focus on standardized, multi-country epidemiological surveillance; large-scale molecular characterization of circulating *Echinococcus* strains; elucidation of wildlife-mediated transmission pathways; and the development of region-specific prevention and control interventions. Local risk factors should be investigated to establish a foundation for precise prevention and control [108]. Diagnostic methods need to be optimized to increase detection accuracy and ease of use. Furthermore, strengthened cross-border collaboration and harmonized surveillance systems are essential to improve data comparability and guide targeted public health actions.

In summary, this review highlights the ongoing public health importance of CE in Africa and highlights urgent research and implementation gaps. Addressing these challenges will enhance our understanding of CE epidemiology and support evidence-based strategies for disease prevention and control across the continent.

## Data Availability

No new data were created or analyzed in this study. Data sharing is not applicable to this article.
